# A Stimulatory Role for Cytokinin in the Arbuscular Mycorrhizal Symbiosis of Pea

**DOI:** 10.3389/fpls.2019.00262

**Published:** 2019-03-12

**Authors:** Dane M. Goh, Marco Cosme, Anna B. Kisiala, Samantha Mulholland, Zakaria M. F. Said, Lukáš Spíchal, R. J. Neil Emery, Stéphane Declerck, Frédérique C. Guinel

**Affiliations:** ^1^Biology, Wilfrid Laurier University, Waterloo, ON, Canada; ^2^Mycology, Applied Microbiology, Earth and Life Institute, Université catholique de Louvain, Louvain-la-Neuve, Belgium; ^3^Biology, Trent University, Peterborough, ON, Canada; ^4^Centre of the Region Haná for Biotechnological and Agricultural Research, Palacký University Olomouc, Olomouc, Czechia

**Keywords:** AM symbiosis, cytokinin, INCYDE, legume, PI-55, *Pisum sativum* L., plant hormone, *Rhizophagus irregularis*

## Abstract

The arbuscular mycorrhizal (AM) symbiosis between terrestrial plants and AM fungi is regulated by plant hormones. For most of these, a role has been clearly assigned in this mutualistic interaction; however, there are still contradictory reports for cytokinin (CK). Here, pea plants, the wild type (WT) cv. Sparkle and its mutant E151 (*Pssym15*), were inoculated with the AM fungus *Rhizophagus irregularis*. E151 has previously been characterized as possessing high CK levels in non-mycorrhizal (myc^-^) roots and exhibiting high number of fungal structures in mycorrhizal (myc^+^) roots. Myc^-^ and myc^+^ plants were treated 7, 9, and 11 days after inoculation (DAI) with synthetic compounds known to alter CK status. WT plants were treated with a synthetic CK [6-benzylaminopurine (BAP)] or the CK degradation inhibitor INCYDE, whereas E151 plants were treated with the CK receptor antagonist PI-55. At 13 DAI, plant CK content was analyzed by mass spectrometry. The effects of the synthetic compounds on AM colonization were assessed at 28 (WT) or 35 (E151) DAI via a modified magnified intersections method. The only noticeable difference seen between myc^-^ and myc^+^ plants in terms of CK content was in the levels of nucleotides (NTs). Whereas WT plants responded to fungi by lowering their NT levels, E151 plants did not. Since NTs are thought to be converted into active CK forms, this result suggests that active CKs were synthesized more effectively in WT than in E151. In general, myc^+^ and myc^-^ WT plants responded similarly to INCYDE by lowering significantly their NT levels and increasing slightly their active CK levels; these responses were less obvious in BAP-treated WT plants. In contrast, the response of E151 plants to PI-55 depended on the plant mycorrhizal status. Whereas treated myc^-^ plants exhibited high NT and low active CK levels, treated myc^+^ plants displayed low levels of both NTs and active CKs. Moreover, treated WT plants were more colonized than treated E151 plants. We concluded that CKs have a stimulatory role in AM colonization because increased active CK levels were paralleled with increased AM colonization while decreased CK levels corresponded to reduced AM colonization.

## Introduction

The biological association between land plants and AM fungi is one of the most ancient, widespread, and functionally important symbioses on Earth. Its development can be dissected into several steps reflecting the progression of the fungus during colonization of the host roots (Smith and Read, 2008). In the pre-contact step, the hyphae growing from a propagule (i.e., spores, vesicles, colonized roots) often start branching to increase the number of potential entry points into the roots (Akiyama et al., 2005). Once in contact with the root surface, the hyphae differentiate into hyphopodia to facilitate fungal penetration into the plant epidermis. In the *Arum*-type of AM colonization, which is the most studied type, the fungal hyphae proliferate intercellularly within the root cortex and eventually invade cortical cells to develop into arbuscules (Dickson et al., 2007), which are the main interfaces in the bi-directional exchange of nutrients between the two partners. While the fungus provides phosphorus, nitrogen, and other mineral elements to the plant (Rausch et al., 2001; Harrison et al., 2002; Guether et al., 2009; Gaude et al., 2012), the plant delivers sugars and lipids to the fungus (Helber et al., 2011; Bravo et al., 2017). Newly acquired carbon is translocated through the hyphae mostly in lipid forms, which are either used in fungal metabolism or stored in spores and other fungal structures, such as vesicles and auxiliary cells depending on the genus.

The development of AM symbiosis involves a complex molecular dialogue between partners and is partially regulated by the host plant through the highly specific action of several hormones. Regulatory aspects of most of the hormones in this process have been discussed in several reviews (Foo et al., 2013; Bucher et al., 2014; Fusconi, 2014; Gutjahr, 2014; Pozo et al., 2015; Lace and Ott, 2018; Liao et al., 2018; Das and Gutjahr, 2019), wherein the role of CKs appears noticeably obscure.

**FIGURE 1:**
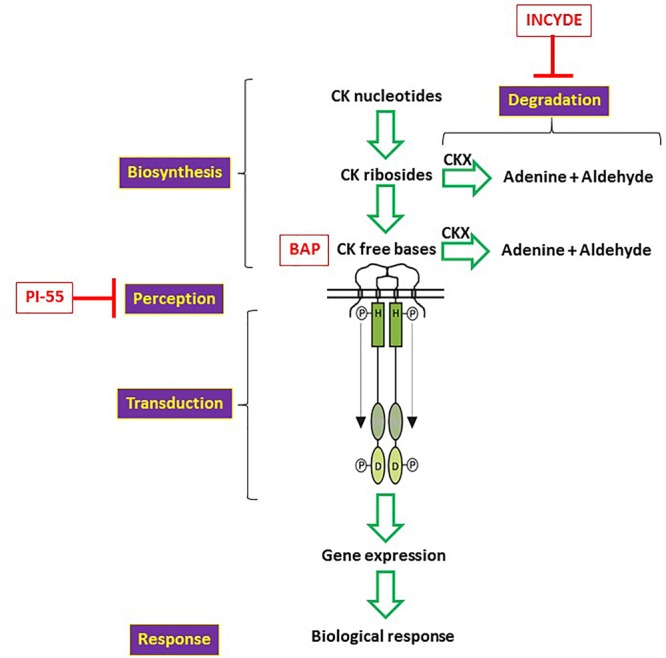
Schematic representation of CK homeostasis in a plant cell. CK nucleotides are the precursors of the CK ribosides and CK free bases (e.g., BAP). Both types of CKs are substrates of the enzyme CK oxidase (CKX) and they will be degraded upon enzymatic activity. When active free bases are perceived by CK receptors, a signal is transduced to the nucleus triggering the expression of response regulators, which ultimately will lead to a biological response. INCYDE, an inhibitor of CK degradation, and PI-55, a competitive inhibitor of CK action, are capable of regulating CK homeostasis.

CKs can exist in various forms that differ in their structure and activity, and their homeostasis is fine-tuned through biosynthesis and degradation (Figure 1; Spíchal, 2012). They are perceived by receptors which consist of an extracellular CHASE domain and an intracellular portion made up of a His kinase domain and two receiver domains (Kieber and Schaller, 2010). Upon CK perception, a signal is transduced and decrypted, and a biological response ensues (Figure 1). It still remains controversial whether CKs have a role in the establishment and maintenance of AM colonization. A regulatory role for CKs is either considered not well understood (Pozo et al., 2015; Liao et al., 2018), continues to cast doubts (Foo et al., 2013; Lace and Ott, 2018; Das and Gutjahr, 2019), or is just overlooked (Bucher et al., 2014; Gutjahr, 2014), possibly because of the contradictory conclusions documented in the literature. For instance, mycorrhizal colonization did not appear to alter gene expression in the CK signaling of the legume species *Medicago truncatula* (Laffont et al., 2015). Yet, recent studies employing powerful biochemical tools (Adolfsson et al., 2017; Schmidt et al., 2017; Yurkov et al., 2017) confirmed what earlier studies had documented (e.g., Allen et al., 1980; Shaul-Keinan et al., 2002), i.e., that mycorrhizal (myc^+^) plants exhibit altered CK levels compared to non-mycorrhizal (myc^-^) plants. The intensity of these mycorrhizal effects on CK accumulation in plants depends on the fungal species, P availability in soil, as well as on plant tissue and development stage.

Several hypotheses have been proposed to explain the AM-mediated changes in CK content of the host plants. First, the AM fungus may produce and deliver CKs to the host roots (Allen et al., 1980; Shaul-Keinan et al., 2002). Second, the AM fungus may stimulate host plant CK biosynthesis (Drüge and Schönbeck, 1992). Third, either a plant or a fungal compound may inhibit CK degradation (Allen et al., 1980; Shaul-Keinan et al., 2002). Finally, the AM-mediated increase in P uptake may indirectly affect the plant CK homeostasis (Drüge and Schönbeck, 1992; Shaul-Keinan et al., 2002). Regardless of the mechanism, considering the strong impact of CKs on plant development (e.g., Werner and Schmülling, 2009), it is likely that alteration of CK levels in the host plant affects AM symbiosis. Yet, the effects that CKs have on AM development appear inconsistent in the few existing reports. For example, the exogenous application of synthetic CK to myc^+^ plants suggests an inhibitory effect of CKs on AM development (Gryndler et al., 1998; Bompadre et al., 2015; Schmidt et al., 2017), whereas both endogenous CK deficiency (Cosme and Wurst, 2013) and elevated CK levels (Jones et al., 2015) in the host plant hint at a stimulatory role. Some of these inconsistencies might be explained by the fact that CKs, depending on their location (shoots or roots), may have distinct roles in the AM symbiosis (Cosme et al., 2016).

Here, we investigated (1) whether the presence of an AM fungus (*Rhizophagus irregularis*) affects the CK levels in the shoots and roots of a legume host plant and (2) whether altering the CK homeostasis of this legume influences AM development. To tackle these two objectives, we used pea (*Pisum sativum* L.) cv. Sparkle (WT) and its pleiotropic mutant E151 (*Pssym15*) as host plants. This mutant was characterized as sparsely forming nodules when grown in the presence of rhizobia (Kneen et al., 1994). Early in its development, i.e., 6 days after planting, E151 is known to accumulate a larger amount of CKs than its WT. Furthermore, later in life, E151 exhibits enhanced mycorrhizal colonization under the control of its shoot as determined by grafting experiments (Jones et al., 2015). In this work, we studied AM development in both these plant lines along several time points and analyzed the CK content of myc^+^ and myc^-^ plants 13 DAI, when pea is known to have entered its vegetative phase (Knott, 1987). To manipulate CK homeostasis, we undertook pharmacological treatments using synthetic compounds modifying the CK status of the plant. Finally, we used bi-compartmented *in vitro* root organ cultures to test if these CK status-modifying compounds have direct effects on the AM fungus development. Overall, our results suggest that fluctuations in CK homeostasis affect significantly AM development in pea. At an early stage of the interaction, a decrease in plant CK NTs, reflecting a fast turnover rate of these precursor molecules into active CKs, facilitated the fungal entry into the roots, while at a later stage high levels of active CKs in the shoot stimulated intraradicular fungal progression.

## Materials and Methods

### Fungal and Plant Growth Conditions

The AM fungal strain used in this study was *R. irregularis* [(Blaszk, Wubet, Renker, and Buscot) C. Walker and Schuessler 2010 as (“irregulare”)] DAOM 197918 originally obtained from the Agriculture and Agri-Food Canada Glomeromycota *in vitro* collection (AAFC, Ottawa, ON, Canada) and propagated in our lab using leek (*Allium ampeloprasum*) as a host. The leek plant cultures with the AM fungus were grown in peat and Turface^®^ [1:1, v:v; ASB Greenworld Ltd. (Mount Elgin, ON, Canada) and Plant Products Company Ltd. (Brampton, ON, Canada), respectively]. They were kept in a growth chamber (16/8 h, 23/18°C, light/dark cycle) and watered weekly. Every 3 weeks, they received a low phosphorus Long-Ashton nutrient solution (Audet and Charest, 2010) instead of water. Roots of leek plants containing hyphae, vesicles and spores of the AM fungus were cut in small fragments. These fragments were mixed with the soil in which the leeks had been grown and together were used as an inoculum source for the growth-room experiments described below. For the *in vitro* experiment, spores and mycelium of *R. irregularis* MUCL 41833 as well as the Ri T-DNA transformed carrot (*Daucus carota*) roots were obtained from the Glomeromycota *in vitro* collection (GINCO^1^), where starting inocula are maintained and distributed, under *in vitro* conditions, in modified Strullu-Romand (MSR) medium.

Seeds of *P. sativum* L. cv. Sparkle and of its mutant E151 were surface-sterilized with 8% bleach, and left imbibing in water overnight in the dark. They were then sown in black Cone-tainers^TM^ (600 mL, Stuewe and Sons, Inc., Tangent, OR, United States) filled with a substrate mixture [1:1:1, v:v:v] of peat: Turface^®^: vermiculite (Therm-O-Rock East Inc., New Eagle, PA, United States). The substrate mixture was autoclaved to eliminate any potential microbial contaminants. For the myc^+^ plants, the fungal inoculum was added at a ratio of 1:5 (v:v) to the sterile substrate mixture before planting. All plants were kept in a growth room (16/8 h, 23/18°C, light/dark regime). For the first 10 days, seedlings were watered when needed. Once established, except when noted otherwise, all plants were subjected to a cycle of water, water, and low phosphorus Hoagland nutrient solution (Resendes et al., 2001), until they were harvested. Harvest time was variable depending on the experiments. Plants used for CK analysis were harvested 13 DAI while plants in the AM development study were harvested 5, 10, 15, 20, and 25 DAI. An initial delay of fungal entry was observed into E151 roots compared to the WT roots (see below section E151 Delays Fungal Entry but Accelerates Intraradical Fungal Development); therefore, WT and E151 myc^+^ plants treated with the synthetic CK status-modifying compounds were harvested at 28 and 35 DAI, respectively. This was considered acceptable because the size of the root systems of 35 day-old E151 plants was similar to that of 28 day-old WT plants. Both treated myc^+^ plant lines were analyzed in comparison to either their respective myc^-^ controls or their respective non-treated myc^+^ controls. For a better visualization of the chronology of events, the timeline of the CK and AM experiments is included as Supplementary Figures 1A,B, respectively.

### Assessment of AM Colonization in Plants

To evaluate the colonization of roots by the AM fungi, root systems were cleaned at harvest, and lateral roots from each plant were randomly selected. They were then cut into 3 cm-segments, and stained for AM structures with Indian ink (Vierheilig et al., 1998). Root segments were mounted onto microscope slides using 60% aqueous glycerol. The magnified intersections method (McGonigle et al., 1990) modified by MacColl (2017) was used to assess AM colonization, yielding estimates of the percentage of root length colonized by hyphae, arbuscules, and vesicles. Briefly, the modification consisted of assessing 105 intersections per slide (15 passes were made over seven roots). Each time an arbuscule or a vesicle was scored, a hypha was scored as well. Root sections were observed under a Zeiss Axiovision light microscope at a 200× magnification (20× objective; 10× ocular).

### Treatment of Plants With Synthetic Compounds Modifying CK Status

To alter plant endogenous CK status, 7, 9, and 11 DAI, myc^+^ and myc^-^ seedlings received several synthetic compounds (Supplementary Table 1). Because 6-day-old WT plants had reduced CK levels compared to the E151 mutant (Jones et al., 2015), the WT plants were treated with 0.1 μM 6-benzylaminopurine (BAP) or with 1 μM 2-chloro-6-(3-methoxyphenyl)-aminopurine (INCYDE; Zatloukal et al., 2008) to increase their CK levels. As well, E151 plants were treated with 10 μM 6-(2-hydroxy-3-methylbenzyl)-aminopurine (PI-55; Spíchal et al., 2009) to reduce their endogenous CK sensing and mimic the effect of CK deficiency. In addition, to confirm the role of PI-55 as a competitor for plant CK receptors, the E151 mutant was treated with either 1 μM BAP or with a mixture of PI-55 (5 mL of 10 μM) and BAP (5 mL of 1 μM). On each day of treatment, plants received at their crown 10 mL of the chemical aqueous solution [with a minimal concentration of the solvent DMSO for INCYDE and PI-55 (0.002 and 0.1%, respectively)].

### CK Extraction, Purification, and Quantification by HPLC-(+ESI)-MS/MS

To assess whether the CK levels were altered by the synthetic compounds modifying the plant CK status, both myc^-^ and myc^+^ plants were harvested 13 DAI. Throughout their life, these plants were provided only with water, i.e., they were never exposed to mineral nutrient fertilization. The roots were immediately separated from the shoots and carefully cleaned. For the aboveground plant tissue sampling, the entire leaf positioned on the fourth node was collected from each plant and approximately 0.1 g of fresh leaf tissue was freeze-dried. For the belowground plant tissue sampling, a total fresh weight of 0.2 g of root tissue per plant was sampled. For this, the fourth lateral root was detached and weighed; in the case when more root tissue was required, the lateral root positioned just below (in terms of vascular pole positioning) was also sampled.

Freeze-dried samples were homogenized in cold (-20°C) modified Bieleski No. 2 extraction buffer [CH_3_OH : H_2_O : HCO_2_H (15:4:1, v:v:v)] using a ball mill grinder and ZnO_2_ beads (25 Hz, 2 min, 4°C, Retsch MM300, Haan, Germany). Internal standards were added to each sample to enable endogenous hormone quantification through isotope dilution. The following standards were added (10 ng of each): [^2^H_7_]BAP, [^2^H_7_]BAPR, [^2^H_5_]ZOG, [^2^H_7_]DHZOG, [^2^H_5_]ZROG, [^2^H_7_]DHZROG, [^2^H_6_]iP7G, [^2^H_5_]Z9G, [^2^H_5_]2MeSZ, [^2^H_6_]2MeSiP, [^2^H_5_]2MeSZR, [^2^H_6_]2MeSiPR, [^2^H_6_]iPR, [^2^H_5_]ZR, [^2^H_3_]DHZR, [^2^H_6_]iP, [^2^H_3_]DHZ, [^2^H_5_]Z, [^2^H_6_]iPRMP, [^2^H_6_]ZRMP, [^2^H_3_]DHZRMP (OlChemIm Ltd., Olomouc, Czechia). CK extraction and purification were carried out as described in Quesnelle and Emery (2007) and modified by Farrow and Emery (2012).

High-performance liquid chromatography-positive electrospray ionization tandem mass spectrometry [HPLC-(+ESI)-MS/MS] was conducted according to Farrow and Emery (2012). Briefly, samples were analyzed using a Shimadzu LC (Kyoto, Japan) connected to a 5500 QTrap triple quadrupole mass spectrometer (Sciex Applied Biosystem, Concord, ON, Canada) with a turbo V-spray ionization source. A 20 μL sample was injected onto a reverse phase C18 column (Kinetex 2.6u C18 100 A, 2.1 × 50 mm; Phenomenex, Torrance, CA, United States). The samples were analyzed in a positive-ion mode. All hormone fractions were eluted with component A: H_2_O with 0.08% CH_3_CO_2_H and component B: CH_3_CN with 0.08% CH_3_CO_2_H, at a flow rate of 0.4 mL min^-1^. The CK fractions were eluted with a multistep gradient. Starting conditions were 5% B increasing linearly to 10% B over 2 min followed by an increase to 95% B over 6.5 min; 95% B was held constant for 1.5 min before returning to starting conditions for 5 min.

All data were analyzed with Analyst 1.5.1 software (AB SCIEX, Concord, ON, Canada). CK types were identified based on their multiple reaction monitoring channels and retention times. CK levels were determined according to isotope dilution analysis via direct comparison of the endogenous analyte peak area to that of the recovered internal standard. The analysis of *cis*Z types was done relative to both the recovery of labeled *trans*Z types and the retention time of unlabeled *cis*Z CKs (Farrow and Emery, 2012).

### Treatment of the AM Fungus With Synthetic CK-Related Compounds Using *in vitro* Root Organ Culture

To understand whether the synthetic CK status-modifying compounds can have direct effects on the growth of the AM fungus, we used *in vitro* bi-compartmented Petri plates containing root organ cultures in one of the compartments (referred to hereafter as the root compartment). The root compartment was filled with MSR medium (Declerck et al., 1998; using Phytagel^TM^ instead of Bacto^TM^ agar as gelling agent). The opposite compartment (hereafter referred to as the HC) was filled with a modified MSR medium, which did not contain sucrose and vitamins (the absence of sucrose and vitamins minimizes potential invasion of HC by roots). Fresh fragments of host roots, i.e., transformed carrot roots, were placed into each root compartment, and inoculated with approximately 400 spores accompanied by hyphal fragments of *R. irregularis* MUCL 41833. All plates were incubated in the dark at 27°C. The plates were checked regularly, and any root tips attempting to cross the compartment barrier were trimmed and removed. In contrast, the AM fungus was allowed to migrate and grow into the HC. Because of the variability between plates of the initial time at which hyphae would invade the HC, the fungus was first left to proliferate in this compartment. Then, the medium in the HC of each plate was removed with a sterile spatula, and the HC was re-filled with the modified MSR medium, this time enriched with 10 μM of either INCYDE, PI-55, or BAP in DMSO solution (0.1%), or with a DMSO solution as a negative control. Each solution had been previously sterilized through a syringe filter (0.2 μm). Afterward, the AM fungus was allowed to regrow into the HC in the presence of the synthetic CK status-modifying compounds or DMSO. We focused on the fungal regrowth to assure that the hyphae in the HC had a similar starting point between plates, and importantly, were exposed to the different synthetic compounds for a similar period. After 35 days of fungal regrowth, the hyphal length density in the HC was determined using the grid-line intersect method (Giovannetti and Mosse, 1980) adapted to determine hyphal length.

### Statistical Analysis

Mean values and standard errors were generated for all data sets and were subjected to different statistical tests depending on the data. Student’s *t*-test (or Mann-Whitney *U*-test in the event of non-normally distributed data) was used to compare: (1) the fungal colonization of WT and E151 roots at different developmental stages; and (2) the CK levels of myc^+^ and myc^-^ pea lines treated with the same synthetic CK status-modifying compound. A one-way analysis of variance (ANOVA) test was used to compare: (a) the *in vitro* hyphal length density treated or not with CK status-modifying compounds; (b) the CK levels measured in both WT and E151 plants with a similar mycorrhizal status; and (c) E151 plants treated or not with CK status-modifying compounds. Each ANOVA was followed by Duncan’s *post hoc* test for (a and b), and a Tukey’s HSD *post hoc* test for (c). To compare the fungal colonization of WT plants treated or not with CK status-modifying compounds, a Kruskal–Wallis test was used followed by a multiple comparison test. Finally, a linear regression was performed to determine whether the levels of putatively active CKs (PACKs; equaled to the sum of the RB and FB levels) is a significant predictor of fungal colonization. All tests were conducted using R Studio (R Core Team, 2017).

## Results

### E151 Delays Fungal Entry but Accelerates Intraradical Fungal Development

**FIGURE 2:**
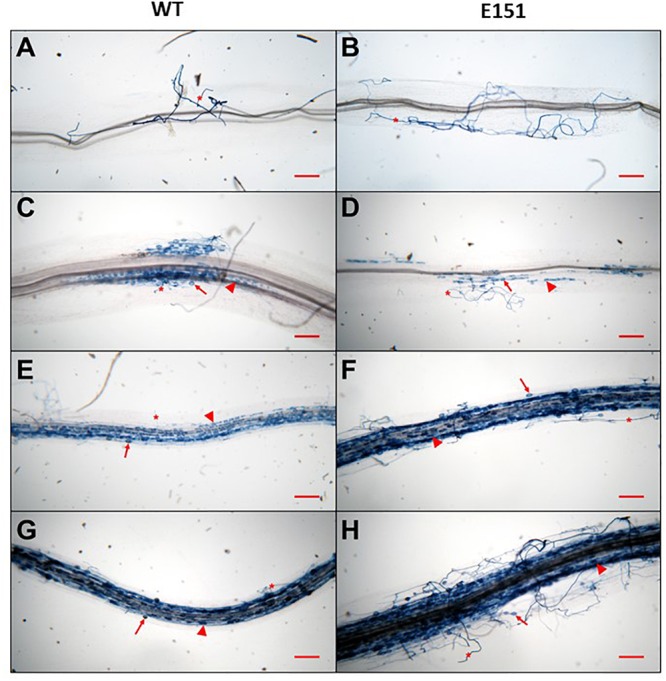
Development of arbuscular mycorrhizae resulting from the association formed between *Rhizophagus irregularis* and pea plants. WT **(A,C,E,G)** and the mutant E151 **(B,D,F,H)** were compared at different time points. At 10 DAI **(A,B)**, extra-radicular hyphae (asterisks) were seen on the root surface of both pea lines. Root entry and subsequent internal colonization of the fungus were first observed at 15 DAI **(C,D)**, at which point fungal colonization in WT appears to be more extensive than in E151. At 15 DAI, arbuscules (arrowheads) and vesicles (arrows) were already formed. At 20 DAI **(E,F)** and 25 DAI **(G,H)**, E151 roots exhibited many more fungal structures than WT roots. Bars = 250 μm.

**FIGURE 3:**
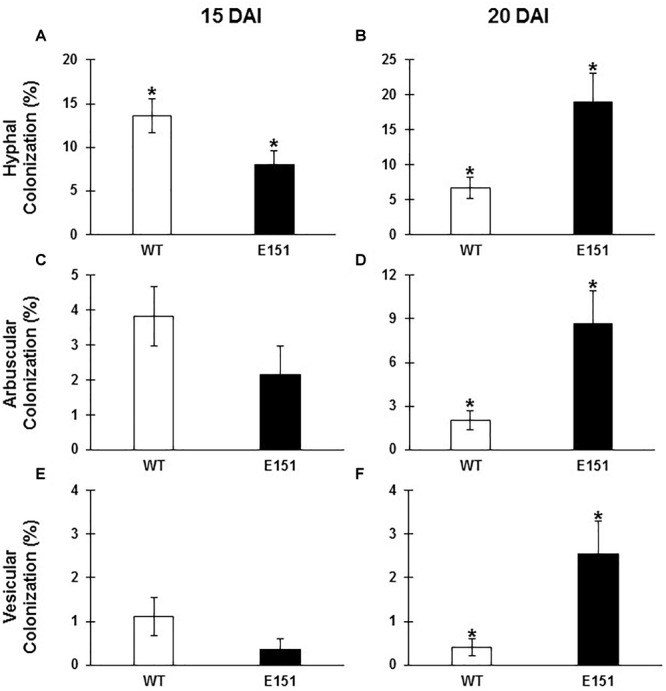
Percent of WT (empty bars) and E151 (solid bars) roots colonized by *R. irregularis*, as determined by assessing percentages of intraradicular hyphae **(A,B)**, arbuscules **(C,D)**, and vesicles **(E,F)** observed at 15 **(A,C,E)** and 20 **(B,D,F)** DAI. These data (mean ± SE; *n* = 5 or 6) are the result of one replicate and are the best representative of three replicates; they were subjected to Student’s *t*-test to determine significant differences between pea lines. An asterisk indicates significance at a 95% confidence level.

There were obvious qualitative (Figure 2) and quantitative (Figure 3) differences between the two pea lines throughout the development of the AM association. Five DAI, there were no clear signs of fungal presence on the roots of either pea line. The earliest visible interaction between *R. irregularis* and the pea plants occurred 10 DAI (Figure 2A,B). At this time, extraradical hyphae ran along the root surface, but did not appear to penetrate the roots. Quantitatively, only half of the sampled WT roots were colonized at 10 DAI, and each colonization unit [i.e., infection unit as per Franson and Bethlenfalvay (1989)] had on average two hyphopodia. There were neither visible hyphopodia nor colonization units on the roots of the E151 mutant line at that time. At 15 DAI, hyphopodia had formed on both pea lines (Figure 2C,D); there were 3.6 ± 0.83 and 2.88 ± 0.73 hyphopodia per colonization unit on the WT and E151 plants, respectively. Although this difference in hyphopodia numbers between lines was not significant, it was associated with a significant increase (Student’s *t*-test; *P* ≤ 0.05) in the percentage of root length colonized by the AM fungus in the WT plants, compared with that of the E151 plants (Figure 3A,C,E). At 20 DAI and later, an obvious shift in AM fungal colonization was observed, with the root cortex of E151 plants being more colonized than that of WT plants (Figure 2E–H). This qualitative increase in fungal colonization of the root cortex at 20 DAI in E151, compared with that of its WT, was quantitatively significant (Student’s *t*-test; *P* ≤ 0.05) for all measured fungal structures (Figure 3B,D,F). In a later stage of fungal colonization, i.e., at 25 DAI, mycorrhizal levels tended to be higher in E151 than in WT, but the difference was not significant (data not shown). Overall, fungal entry into the roots appeared to be delayed in E151. However, once the fungus was established in the cortex of E151, its intraradical proliferation was accelerated in terms of hyphal growth as well as of fungal differentiation into arbuscules and vesicles, when compared with that of the WT.

### Mycorrhizal WT Plants Exhibit ReducedEndogenous CK Nucleotide Levels at 13DAI While E151 Plants Do Not

To understand how the AM fungus affected the CK profiles of WT and E151 pea plants, we measured the CK levels in shoots and roots of both myc^-^ and myc^+^ plants at 13 DAI. The mycorrhizal status (myc^-^ and myc^+^) of the plants was confirmed at harvest by assessing fungal colonization (absence/presence, respectively).

**FIGURE 4:**
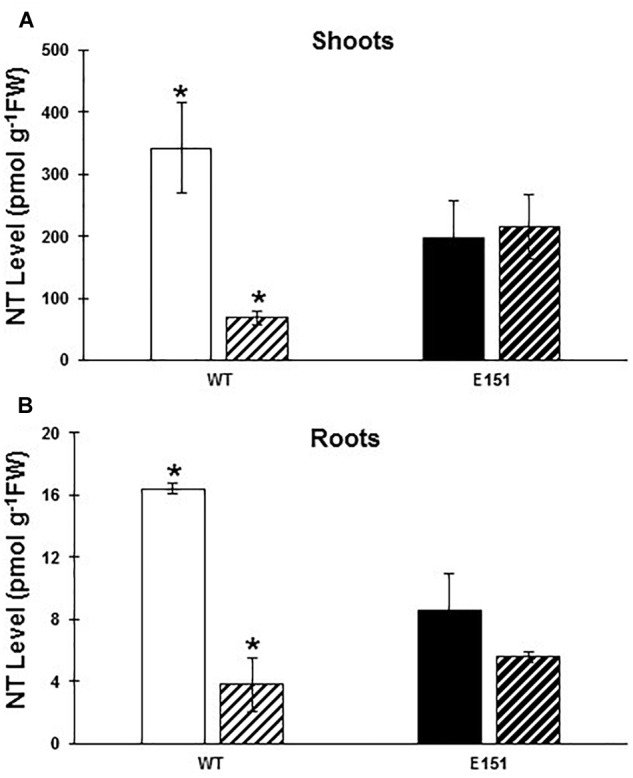
Levels [pmol g^-1^ of fresh weight (FW)] of nucleotides (NT; *trans*ZNT, *cis*ZNT, and iPNT) in the shoots **(A)** and the roots **(B)** of WT (white bars) and E151 (black bars) plants. Plants inoculated with *R. irregularis* (bars with hatched lines) or non-inoculated (solid bars) were harvested 13 days after planting. Data (mean ± SE; *n* = 3) were subjected to a Student’s *t*-test to determine significant differences between myc^-^ and myc^+^ plants. An asterisk indicates significance at a 95% confidence level.

Although no obvious differences were detectable in the total CK levels between myc^-^ WT and myc^-^ E151 plants in either shoots or roots (Table 1), the response of E151 differed markedly from its WT counterpart in terms of the fluctuations observed in the NT levels following AM colonization (Figure 4). Whereas the levels of the NT fraction in WT plants were reduced significantly (Student’s *t*-test; *P* ≤ 0.05) in both shoots (Figure 4A) and roots (Figure 4B) in response to the AM fungus, the levels of the NT fraction in both shoots and roots of E151 plants remained mostly unaltered. Among all NT compounds detected, i.e., *trans*ZNT, *cis*ZNT and iPNT, the most abundant form in the shoots of WT and E151 plants was iPNT (Table 2). In WT shoots, iPNT levels were significantly reduced (Student’s *t*-test; *P* ≤ 0.05) following AM colonization, while the levels of *trans*ZNT and *cis*ZNT were moderately reduced (Table 2). In contrast, in the roots of WT and E151, the levels of iPNT were below the detection limit, while the most abundant NT compound was *cis*ZNT (Table 3). After colonization, the levels of *cis*ZNT were significantly reduced (Student’s *t*-test; *P* ≤ 0.05) in WT roots, while the levels of *trans*ZNT remained almost unaltered (Table 3). The levels of NT in roots of E151 decreased slightly following colonization; however, the observed changes were not significant.

**Table 1:** Levels [pmol g^-1^ of fresh weight (FW)] of total CK isolated from shoots and roots of non-mycorrhizal (myc^-^) and mycorrhizal (myc^+^) pea plants.

	Total CK level (pmol g^-1^FW)			
Treatment	Shoots		Roots	
	myc^-^	myc^+^	myc^-^	myc^+^
WT control	1028.31 ± 244.18^a^	915.61 ± 81.29^a^	24.40 ± 3.30^a^*	8.74 ± 1.00^a^*
WT + BAP	1074.14 ± 59.72^a^	932.95 ± 37.15^a^	14.59 ± 3.53^ab^	13.08 ± 2.60^a^
WT + INCYDE	1204.05 ± 232.30^a^	1208.59 ± 61.30^a^	11.34 ± 2.98^b^	11.06 ± 3.24^a^
E151 control	847.79 ± 50.05^ab^	1019.19 ± 190.30^a^	14.93 ± 3.20^ab^	11.26 ± 1.62^a^
E151 + PI-55	492.15 ± 52.22^b^	522.12 ± 97.58^b^	11.40 ± 2.23^b^	8.89 ± 0.80^a^

*CK content was measured in wild type (WT) plants, in WT plants treated with BAP and INCYDE, in the pea mutant E151, and in E151 plants treated with PI-55. Values are means ± SE (*n* = 3). Different letters indicate a significant difference between treatments (one-way ANOVA followed by a Duncan’s *post hoc* test, 95% confidence level). An asterisk indicates a significant difference between the same treatments of differing mycorrhizal status (Student’s *t*-test, 95% confidence level).*

**Table 2:** Levels [pmol g^-1^ of FW] of CK isolated from shoots of non-mycorrhizal (myc^-^) and mycorrhizal (myc^+^) pea plants.

Pea line + Treatment	Cytokinin level [pmol g^-1^FW]							
	Free bases (FB)	Ribosides (RB)				Nucleotides (NT)		
	iP	DHZR	transZR	cisZR	iPR	transZNT	cisZNT	iPNT
	**myc^-^**							
WT control	5.30 ± 0.78^ab^	2.81 ± 0.42^ab^	0.70 ± 0.29^a^	172.16 ± 54.38^ab^	211.55 ± 44.14^ab^	1.01 ± 0.05	42.93 ± 13.67	298.47 ± 45.58^a^*
WT+BAP	9.44 ± 0.21^b^	3.10 ± 0.04^ab^	1.91 ± 0.16^b^	178.64 ± 12.08^ab^	303.89 ± 22.34^b^	0.82 ± 0.07	16.75 ± 1.13*	165.00 ± 18.31^ab^
WT+INCYDE	8.07 ± 1.03^b^	3.39 ± 0.33^a^	1.58 ± 0.14^bc^	215.74 ± 10.03^a^	317.62 ± 28.39^b^	0.80 ± 0.01	13.79 ± 3.90	129.42 ± 44.34^b^
E151 control	5.89 ± 1.94^ab^	2.27 ± 0.59^ab^	1.18 ± 0.18^abc^	122.41 ± 38.39^ab^	201.35 ± 77.09^ab^	1.43 ± 0.38	26.91 ± 8.84	170.14 ± 38.88^ab^
E151+PI-55	1.68 ± 0.33^a^	1.72 ± 0.01^b^	1.09 ± 0.07^ac^	60.09 ± 9.62^b^	54.70 ± 15.28^a^	1.22 ± 0.15	45.29 ± 11.89	241.53 ± 23.21^ab^*
	**myc^+^**							
WT control	7.05 ± 1.31^ab^	2.64 ± 0.10	1.37 ± 0.12^ab^	149.63 ± 19.27^ab^	269.24 ± 25.47^b^	0.89 ± 0.24	8.24 ± 0.32	60.04 ± 9.34^b^*
WT+BAP	7.62 ± 1.96^ab^	2.51 ± 0.30	1.32 ± 0.31^ab^	147.36 ± 33.61^ab^	238.83 ± 35.37^b^	0.72 ± 0.06	8.43 ± 0.92*	136.77 ± 27.12^ab^
WT+INCYDE	9.71 ± 0.37^a^	2.06 ± 0.61	1.97 ± 0.31^a^	176.79 ± 16.25^a^	340.07 ± 9.29^b^	0.70 ± 0.19	5.09 ± 1.00	45.66 ± 8.82^b^
E151 control	6.24 ± 1.40^ab^	2.16 ± 0.19	1.33 ± 0.18^ab^	148.92 ± 15.16^ab^	265.36 ± 32.44^b^	1.01 ± 0.15	16.03 ± 5.64	198.65 ± 42.32^a^
E151+PI-55	3.20 ± 0.80^b^	1.46 ± 0.12	0.97 ± 0.25^b^	80.21 ± 8.19^b^	117.90 ± 24.79^a^	1.27 ± 0.07	13.34 ± 2.65	129.62 ± 10.63^ab^*

	**Cytokinin level [pmol g^-^^1^FW]**							
	**O- Glucosides (OG)**				**Methyl-thiols (MeS)**			
	**transZROG**		**cisZROG**		**MeSZ**		**MeSZR**	

	**myc^-^**							
WT control	1.79 ± 0.26^ab^		16.83 ± 3.44^bc^		24.59 ± 7.92		250.15 ± 50.96^ab^	
WT+BAP	2.09 ± 0.27^ab^		33.78 ± 1.23^a^*		25.89 ± 2.17		332.82 ± 12.54^b^	
WT+INCYDE	2.28 ± 0.24^a^		32.16 ± 5.53^ac^		25.29 ± 1.78*		453.93 ± 50.42^b^	
E151 control	1.51 ± 0.48^ab^		16.70 ± 5.76^bc^		24.19 ± 6.11		273.80 ± 106.16^ab^	
E151+PI-55	0.88 ± 0.15^b^		5.39 ± 1.64^b^		13.29 ± 4.27		65.27 ± 8.96^a^	
	**myc^+^**							
WT control	1.73 ± 0.18^b^		23.33 ± 1.82		24.31 ± 3.32^b^		367.13 ± 33.96^b^	
WT+BAP	1.65 ± 0.17^ab^		22.12 ± 0.69*		24.78 ± 3.76^b^		340.84 ± 75.54^b^	
WT+INCYDE	2.13 ± 0.23^b^		22.33 ± 3.04		39.88 ± 3.68^a^*		562.21 ± 2.96^a^	
E151 control	1.82 ± 0.21^b^		21.11 ± 4.33		23.02 ± 3.65^b^		333.54 ± 39.67^b^	
E151+PI-55	0.96 ± 0.13^a^		13.79 ± 3.29		11.88 ± 2.19^b^		147.51 ± 35.98^c^	

*CK content was measured in wild type (WT) plants and the pea mutant E151, in WT plants treated with BAP and INCYDE, and in E151 plants treated with PI-55. For CK, Z, DHZ and iP designate zeatin, dihydrozeatin and isopentenyl adenine, respectively. Values are means ± SE (*n* = 3). Different letters indicate a significant difference between treatments (one-way ANOVA followed by a Duncan’s *post hoc* test, 95% confidence level). An asterisk indicates a significant difference between the same treatments of differing mycorrhizal status (Student’s *t*-test, 95% confidence level).*

**Table 3:** Levels [pmol g^-1^ of FW] of CK isolated from roots of non-mycorrhizal (myc^-^) and mycorrhizal (myc^+^) pea plants.

Pea line +	Cytokinin level [pmol g^-1^FW]											
Treatment	Free bases (FB)	Ribosides (RB)				Nucleotides (NT)			O-Glucosides (OG)		Methyl-thiols (MeS)	
	iP	DZR	transZR	cisZR	iPR	transZNT	cisZNT	iPNT	transZROG	cisZROG	MeSZ	MeSZR
	**myc^-^**											
WT control	0.26 ± 0.02	0.34 ± 0.10	0.46 ± 0.12	4.57 ± 1.40^a^	n.d.	0.61 ± 0.03^a^	15.82 ± 1.43^a^*	n.d.	n.d.	2.35 ± 0.19	n.d.	n.d.
WT+BAP	0.17 ± 0.00	0.22 ± 0.03	0.18 ± 0.03*	2.40 ± 0.59^ab^	n.d.	0.41 ± 0.08^ab^	8.84 ± 2.02^b^	n.d.	n.d.	2.37 ± 0.21	n.d.	n.d.
WT+INCYDE	0.20 ± 0.02	0.29 ± 0.03	0.30 ± 0.05	1.67 ± 0.48^ab^	n.d.	0.35 ± 0.03^b^	5.81 ± 2.21^b^	n.d.	n.d.	2.72 ± 0.29	n.d.	n.d.
E151 control	0.24 ± 0.02	0.45 ± 0.11	0.31 ± 0.06	1.83 ± 0.41^ab^	n.d.	0.49 ± 0.07^ab^	8.14 ± 1.92^b^	n.d.	n.d.	3.46 ± 0.33*	n.d.	n.d.
E151+PI-55	0.22 ± 0.05	0.52 ± 0.06	0.26 ± 0.05	1.11 ± 0.36^b^	n.d.	0.44 ± 0.05^ab^	5.96 ± 1.67^b^	n.d.	n.d.	2.90 ± 0.38	n.d.	n.d.
	**myc^+^**											
WT control	0.28 ± 0.05	0.28 ± 0.04	0.55 ± 0.15^b^	2.28 ± 0.54	n.d.	0.54 ± 0.10	3.27 ± 0.29*	n.d.	n.d.	1.55 ± 0.18	n.d.	n.d.
WT+BAP	0.20 ± 0.06	0.32 ± 0.02	0.62 ± 0.06^b^*	2.73 ± 0.11	0.27 ± ± 0.12	0.56 ± 0.10	6.54 ± 1.44	n.d.	n.d.	1.84 ± 0.05	n.d.	n.d.
WT+INCYDE	0.19 ± 0.03	0.27 ± 0.05	0.36 ± 0.05^ab^	2.51 ± 0.70	n.d.	0.59 ± 0.03*	5.40 ± 1.47	n.d.	n.d.	1.73 ± 0.16	n.d.	n.d.
E151 control	0.16 ± 0.03	0.43 ± 0.03	0.41 ± 0.07^ab^	2.38 ± 0.78	0.41 ± ± 0.34	0.72 ± 0.13	4.87 ± 1.45	n.d.	n.d.	1.88 ± 0.24*	n.d.	n.d.
E151+PI-55	0.20 ± 0.03	0.29 ± 0.04	0.21 ± 0.02^a^	1.04 ± 0.23	n.d.	0.88 ± 0.13	4.65 ± 0.17	n.d.	n.d.	1.63 ± 0.17	n.d.	n.d.

*CK content was measured in control plants of wild type (WT) and the pea mutant E151, in WT plants treated with BAP and INCYDE, and in E151 plants treated with PI-55. For CK, Z, DHZ and iP designate zeatin, dihydrozeatin and isopentenyl adenine, respectively. n.d.: not detectable. Values are means ± SE (*n* = 3). Different letters indicate a significant difference between treatments (one-way ANOVA followed by a Duncan’s *post hoc* test, 95% confidence level). An asterisk indicates a significant difference between the same treatments of differing mycorrhizal status (Student’s *t*-test, 95% confidence level).*

The AM fungus did not significantly affect the other CK fractions in either the WT or the E151 mutant (Tables 2, 3), except for the O-glucosides in the roots of E151. Among the O-glucosides measured in the roots (Table 3), the levels of *trans*ZROG were below detection limit, while those of *cis*ZROG were detected in low amounts and revealed a significant reduction (Student’s *t*-test; *P* ≤ 0.05) in myc^+^ E151 roots, compared with that of myc^-^ E151 roots. Overall, the colonization by the AM fungus most evidently reduced the endogenous levels of NTs, an inactive form of CKs, in WT plants but did not affect those of E151 plants.

### CK Promotes the Intraradical Growth of *R. irregularis* but Not the Extraradical Growth

#### Levels of Endogenous CK Can Be Manipulated Pharmacologically in Pea

The effect of the synthetic CK status-modifying compounds on the total CK content of pea plants depended on the organ and the mycorrhizal status of the plant (Table 1). Whereas BAP and INCYDE did not have a significant effect on the total CK levels of the shoots of either myc^-^ or myc^+^ WT plants, the INCYDE treatment significantly reduced (Duncan’s *post hoc* test; *P* ≤ 0.05) the total CK levels of the roots of myc^-^ WT plants. PI-55 treatment tended to decrease CK levels independently of plant organ or fungal presence. This compound had the strongest impact on the total CK content of myc^+^ E151 shoots (Table 1) as it significantly reduced their levels (Duncan’s *post hoc* test; *P* ≤ 0.05). To better understand how these compounds affected the plant CK homeostasis, we compared below the changes observed in different CK fractions (NT, RB, and FB).

**FIGURE 5:**
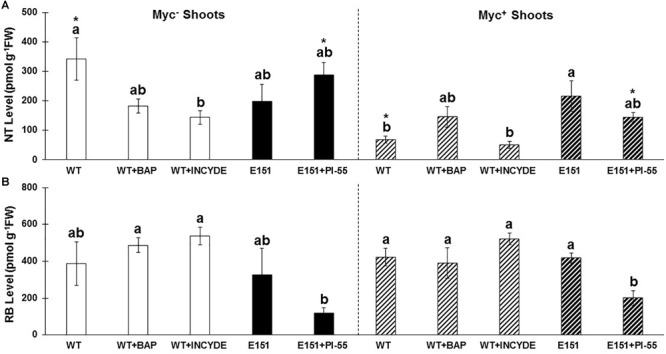
Effects of chemical and mycorrhizal treatments on the levels (pmol g^-1^ of fresh weight) of **(A)** nucleotides (NT; *trans*ZNT, *cis*ZNT, and iPNT) and **(B)** ribosides (RB; DHZR, *trans*ZR, *cis*ZR, and iPR) in the shoots of WT (white bars) and E151 (black bars) plants, the crown of which had been treated with synthetic CK status-modifying compounds. These plants were myc^-^ (solid bars; on the left) or myc^+^ (hatched bars; on the right). Data (mean ± SE; *n* = 3) were subjected to a one-way ANOVA followed by a Duncan’s *post hoc* test (95% confidence level). Different letters indicate significant differences between different chemical treatments of a similar mycorrhizal status. Additionally, Student’s *t*-tests (95% confidence level) were conducted to determine any significant differences, indicated by asterisks, between similar treatments of differing mycorrhizal status within a pea line.

In the shoots and roots of myc^-^ WT plants, NT levels (Tables 2, 3, respectively; Figure 5A) decreased in plants treated with either BAP or INCYDE; specifically, the *cis*ZNT levels were reduced by half or more in the two organs, but the decrease was significant only in the roots (Duncan’s *post hoc* test; *P* ≤ 0.05). Moreover, while the levels of the RBs and FBs tended to increase in the shoots (Table 2 and Figure 5B), they were not affected in the roots of myc^-^ WT plants (Table 3). The NT and RB levels in the shoots of myc^+^ WT plants were affected by the INCYDE treatment in a manner similar to those of shoots in the myc^-^ WT plants (Figure 5A,B and Table 2), but the effect caused by BAP on the myc^+^ plants was not as clear-cut as that of INCYDE (Figure 5). As for the myc^+^ roots of INCYDE- or BAP-treated WT plants, no significant differences were seen when compared to non-treated WT plants, and no obvious trends were seen in the specific CK fractions (Table 3).

Treatment of myc^-^ E151 plants with PI-55 resulted in a noticeable, though non-significant, increase of the shoot NT levels (Figure 5A) and a clear, yet non-significant, decrease of the levels of RBs (Figure 5B) and FBs (Table 2). In the roots of treated myc^-^ E151 plants, the results of all CK fractions tended to decrease (Table 3). Myc^+^ E151 plants responded to the PI-55 treatment differently from myc^-^ E151 plants. In the presence of PI-55, NT levels (Figure 5A) tended to decrease in the shoots of myc^+^ E151 plants compared to those of untreated plants. Moreover, the RB levels (Figure 5B) were significantly reduced (Duncan’s *post hoc* test; *P* ≤ 0.05), likely because of a significant reduction in the iPR fraction (Table 2). As well, the FB levels tended to decrease in the shoots of myc^+^ E151 plants treated with PI-55 (Table 2). The levels of all CK fractions either did not change or tended to slightly decrease in the myc^+^ roots of PI-55-treated E151 plants (Table 3).

In the shoots of both myc^-^ pea lines, the levels of the O-glucoside and methyl-thiol CK fractions, especially their RB conjugates, followed patterns similar to those seen in the RB and FB fractions (Table 2), i.e., they tended to increase in BAP- or INCYDE-treated WT plants and to decrease in PI-55-treated E151 plants. Overall, whereas the effects of BAP and INCYDE treatments on the WT plants were not strongly altered by the presence of the fungus, the effect of PI-55 on the mutant was exacerbated because E151-treated myc^+^ plants exhibited a significant reduction in shoot NT levels compared to the myc^-^ plants (Student’s *t*-test, *P* ≤ 0.05).

#### Endogenous CKs Affect Mycorrhizal Colonization of Plants

**FIGURE 6:**
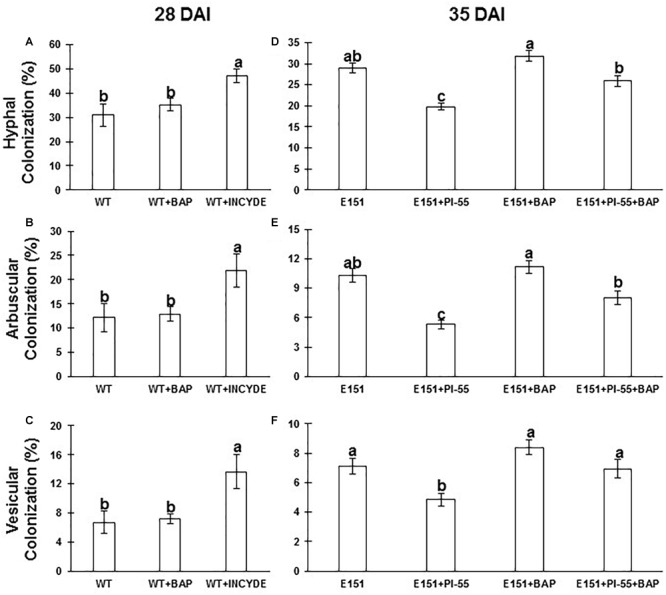
Effect of synthetic CK status-modifying compounds on hyphal colonization **(A,D)**, arbuscular colonization **(B,E)**, and vesicular colonization **(C,F)** in the roots of WT **(A–C)** and E151 **(D–F)** plants inoculated with *R. irregularis*. Whereas WT plants were treated with BAP (0.1 μM) and INCYDE (1 μM), E151 plants were treated with PI-55 (10 μM), BAP (1 μM), and a combination of PI-55 and BAP. WT and E151 plants were harvested 28 and 35 days after inoculation (DAI), respectively. WT data (mean ± SE; *n* = 13, three technical replicates) and E151 data (mean ± SE; *n* = 15; three technical replicates) were either subjected to a one-way ANOVA followed by a Tukey’s HSD *post hoc* test, or a Kruskal–Wallis followed by a multiple comparisons test; significance (95% confidence level) is indicated by different letters.

Altering the endogenous CK levels of the host plants via pharmacological treatments had a significant effect on the intraradicular growth of the AM fungus. When RB and FB levels tended to increase in the WT at 13 DAI, e.g., with INCYDE (Figure 5B), the percentages of root cortex length colonized by hyphae, arbuscules, and vesicles were significantly increased at 28 DAI (multiple comparison test; *P* ≤ 0.05; Figure 6A–C). Moreover, decreasing the RB levels of the mutant E151 by interfering with CK perception via PI-55 treatment (Figure 5B) led to significant reductions (Tukey’s *post hoc* test; *P* ≤ 0.05) in AM fungal colonization of the roots (Figure 6D–F). When the E151 plants were treated with a mixture of BAP and PI-55, the percentages of root length colonized by the AM fungus were intermediate between those obtained with the application of either BAP or PI-55 alone, i.e., those for the intraradical hyphae and arbuscules were significantly lower than the percentages in E151 plants treated only with BAP and significantly higher (Tukey’s *post hoc* test; *P* ≤ 0.05) than the percentages in E151 plants treated only with PI-55 (Figure 6D–F).

**FIGURE 7:**
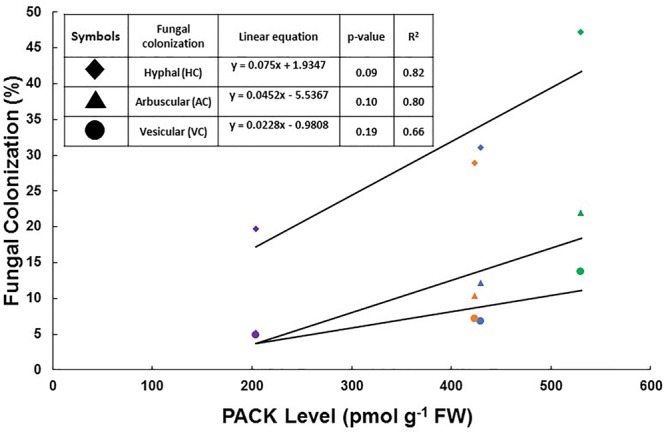
Effect on fungal colonization (%) of the levels of putatively active CK (PACK; sum of riboside and free base fractions) measured in shoots of plants, the crowns of which had been subjected to treatments with synthetic CK status-modifying compounds. Each data-point corresponds to the mean of the fungal colonization assessed in the *R. irregularis*-inoculated plants. The symbols in purple represent the colonization seen in E151 roots treated with PI-55; those in orange represent the colonization seen in non-treated E151 roots. The symbols in blue represent the colonization assessed in non-treated WT roots, and those in green the colonization seen in WT roots treated with INCYDE. This graph is a composite graph created to decipher whether or not a relationship existed between AM fungal colonization and shoot levels of PACK. Whereas the PACK analysis was performed at 13 DAI, the assessment of fungal colonization was done at 28 DAI for WT and at 35 DAI for E151. A linear regression analysis was performed on the distribution of PACK and HC, AC, and VC; the resulting *p*-values and *R^2^* values are shown in a table, along with the linear equation for each fungal structure.

Finally, we tested whether a correlation occurred between AM colonization and PACK levels. We plotted in a single graph the percentages of AM fungal colonization (dependent variable) against PACK levels (independent variable) measured in non-treated WT and E151 plants as well as WT and E151 plants exposed to INCYDE and PI-55, respectively, because both of these treatments were the most effective in altering AM colonization. We did not take into consideration the pea lines or the different times of harvest but considered each treatment identity as a single data-point (Figure 7); as such, we subjected the data to a regression analysis. Although not significant, we observed a positive relationship between the percentages of root length colonized by AM fungal hyphae and the levels of PACK (Figure 7; *R^2^* = 0.82). Likewise, the percentage of root length colonized by the arbuscules and vesicles had an apparent positive relation with the levels of PACK, with *R^2^* values of 0.80 and 0.66, respectively (Figure 7).

#### Synthetic CK Status-Modifying Compounds Have No Effect on the Extraradical Hyphal Growth

To determine whether the synthetic CK status-modifying compounds could directly affect the development of AM fungus, we applied BAP, INCYDE, PI-55, or a DMSO mock solution to the HC of *in vitro* bi-compartmented Petri plates, in which the extraradical hyphae of *R. irregularis* were allowed to grow for 35 days linked to, but separated from, the host roots. A non-significant, minor increase in hyphal length density was measured in the HC to which INCYDE or PI-55 had been added, when compared to that observed in the control HC. As for the hyphal length density in the HC supplemented with BAP, it was similar to that observed in the control HC. Thus, none of the treatments with the synthetic CK status-modifying compounds was able to affect significantly the extraradical fungal growth.

## Discussion

**FIGURE 8:**
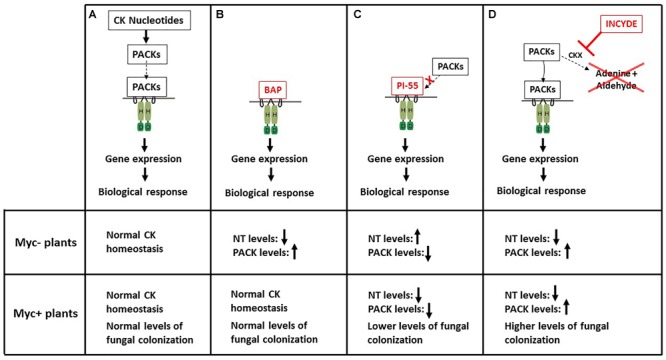
Schematic diagram representing both the perception and the signal transduction pathway of CK in myc^-^ and myc^+^ plants that were either untreated **(A)**, treated with the cytokinin BAP **(B)**, the competitive inhibitor of CK action PI-55 **(C)**, or the inhibitor of CK degradation INCYDE **(D)**. Upon treatment with the synthetic compounds, levels of NTs and of putatively active CKs (PACKs, comprised of both CK ribosides and CK free bases) are altered. An arrow oriented upward corresponds to an increase in CK levels, whereas an arrow facing downward represents a decrease in CK levels. Higher PACK levels correspond to higher AM colonization whereas lower PACK levels are related to lower AM colonization.

Most, and possibly all, plant hormones contribute to the fine-tuning of the development of AM symbiosis in plant roots, and as such they influence the fungal growth required for the exchange of benefits between the symbiotic partners. Amongst the many plant hormones, CKs have one of the least understood roles during AM development (e.g., Pozo et al., 2015; Bedini et al., 2018; Liao et al., 2018). Here, we provide evidence toward a stimulatory role for CKs in AM fungal colonization (Figure 8). On one hand, low levels of NT facilitate fungal entry into the roots; on the other hand, high levels of RBs and the most metabolically active FBs stimulate fungal proliferation within the root cortex. Our findings are strengthened by the use of a pharmacological approach whereby CK homeostasis is altered in myc^+^ plants using different synthetic compounds (Figure 8). Increasing exogenous or endogenous CK levels leads to an increase in AM colonization whereas mimicking CK deficiency results in lower AM colonization.

### Plant Endogenous CK Levels Are Affected by the Presence of the AM Fungi

It is a well-known fact that CK homeostasis is altered in response to AM fungal colonization (e.g., Allen et al., 1980; Baas and Kuiper, 1989; Danneberg et al., 1992; Drüge and Schönbeck, 1992; Goicoechea et al., 1995; van Rhijn et al., 1997; Shaul-Keinan et al., 2002). Among the many studies performed, two are of interest as the CK content was measured at different stages of AM development. While van Rhijn et al. (1997) measured CK in myc^+^ alfalfa plants at 14, 16, and 18 DAI, Yurkov et al. (2017) determined CK content in *Medicago lupulina* at 1, 14, 21, 35, and 50 DAI. In both studies, CK levels were estimated by immunoassay, and thus their results are difficult to compare to ours. It is only recently that researchers have been using the HPLC-ESI MS/MS technique for a precise quantification of CKs and identification of their different forms in myc^+^ plants. Schmidt et al. (2017) working on *Miscanthus* × *giganteus* and Adolfsson et al. (2017) studying *M. truncatula* used plants older than those used in our study (70 DAI and 28 DAI, respectively), and measured the leaf CK content of plants inoculated with *R. irregularis*. In both works, increases in PACK and glucosides (the latter representing CK storage forms) were reported in plants that were well colonized.

To our knowledge, no study has ever reported a decrease in the NT fraction of myc^+^ plants. This is not necessarily surprising since a high disparity exists among the performed studies. Differences in plants species and developmental stages, in fungal species, in growth conditions and in techniques are obvious. For example, in the two most recent studies (Adolfsson et al., 2017; Schmidt et al., 2017), the analytical methods used could not detect NT levels. Another explanation for the NT levels not being reported may be that the NT fraction is not metabolically active; however, any fluctuation in NT levels may indicate, albeit indirectly, changes occurring in the other CK fractions. Thus, high NT levels likely reflect a low turnover into active FBs or RBs, whereas low NT levels could suggest a high conversion into more metabolically active CK fractions (Figure 1).

In most of the previous works, CK levels were shown to be higher in myc^+^ plants than in myc^-^ plants, and the RB fraction appeared to be the most sensitive to the fungal presence. This fraction was higher in roots (e.g., flax in Drüge and Schönbeck, 1992; tobacco in Ginzberg et al., 1998) and shoots (*M. lupulina* in Yurkov et al., 2017) of myc^+^ plants. Furthermore, the changes observed depended on the infection rate (Drüge and Schönbeck, 1992) and the P levels to which the plants were exposed (*Plantago major* in Baas and Kuiper, 1989; leek in Torelli et al., 2000). RBs are considered the main form by which CKs are transported between roots and shoots (Ko et al., 2014; Zhang et al., 2014). In our study, we did not see any differences in the RB levels between myc^-^ and myc^+^ plants and this may be because the CK profiles were analyzed early in the AM symbiosis, i.e., 13 DAI. This result is consistent with that of Dixon et al. (1988), who found that AM development increased RB transport from root to shoot in *Citrus jambhiri*, and that this effect was much weaker early in development (15-day-old) than later, with the strongest effect occurring in 90-day-old plants. It would be logical that RBs are transported in much higher quantities when nutrient exchange is well underway between the two partners of the symbiosis. An increase in CK translocation from the roots to the shoot could be part of the positive feedback thought to take place between AM functioning and shoot CKs (Cosme et al., 2016).

### Pharmacological Treatments of Pea Crowns With Synthetic CK Status-Modifying Compounds Are Effective in Altering Plant CK Homeostasis

In myc^-^ WT plants subjected to either BAP or INCYDE, levels of the NT fraction tended to decrease and those of the PACK fraction to increase (Figure 8A,D). These changes were expected (Aremu et al., 2015) and likely reflect the perception by the treated plants of higher levels of CKs caused by either the exogenous application of BAP or the inhibition of cytokinin dehydrogenases (CKX). As a response to the increased endogenous PACK levels, plants likely down-regulate their CK biosynthesis, leading to a decrease of the CK precursor molecules, i.e., the NT fraction. In myc^-^ E151 plants subjected to PI-55, a synthetic compound known to alter CK perception (Spíchal et al., 2009), the NT fraction tended to increase, whereas RB and FB fractions tended to decrease (Figure 8C). This suggests that, when PI-55 competes with active CK molecules for a CK receptor, a signal is conveyed to the mutant to down-regulate its CK synthesis because it perceives an abundance of CK active forms. Consequently, E151 would accumulate NTs that are not being further transformed. Interestingly, when *Eucomis autumnalis*, a species from the Asparagaceae family, was treated with 10 μM PI-55, no change in the NT levels was observed; however, when it was treated with 0.1 μM PI-55, a large increase in that fraction was noticed (Aremu et al., 2015). These differential effects highlight that responses may depend on the plant species and possibly growth conditions.

We were expecting that INCYDE and BAP would cause a similar outcome for the CK levels of the treated plants, namely that both compounds would increase the endogenous CK levels (Supplementary Table 1), yet there were differences. It is possible that BAP is not as biologically active as the naturally produced, endogenous CKs, the levels of which increased with the INCYDE treatment. Alternately, these differences may be explained by the different fates that these two synthetic CK-related compounds have inside the plant. Whereas INCYDE would directly increase the endogenous CK pool because of the specific inhibition of its target CKX, BAP may be diverted from its site of entry to its site of action (Sakakibara, 2006) and be converted into inactive CK forms such as glucosides along the way. The action may thus be seen as “diluted” or off-target inside the plant (Sakakibara, 2006; Skalický et al., 2018).

### CKs Play a Role in the Development of Mycorrhizae

#### High Levels of Active CKs, as Reflected by Low NT Levels, Are Required for Fungal Entry

At 13 DAI, the levels of the NT fraction in myc^+^ WT plants, in both shoot and root, were significantly lower than those in myc^-^ WT plants, and this drop occurred at a time of fungal entry into the roots (Supplementary Figure 1). In E151 myc^+^ plants, there was no decrease in the NT fraction, and no fungal penetration in the roots was visible. However, treating the mutant with PI-55 led to reductions of both NT levels and AM colonization (Figure 8C). Thus, we hypothesize that, for the fungus to enter a root, it needs its host plant to have a low ratio of non-active CKs to active CKs (hereafter referred to as NT to PACK ratio). Inhibition of fungal entry in E151 is seen in the initial steps of the interaction, and also later in development as shown by Jones et al. (2015) who documented that the fungal hyphopodia were more numerous on E151 roots than on WT roots. These hyphopodia were largely abnormal and septate, which is an indication of retracted fungal cytoplasm and senescence (Glenn et al., 1985), suggesting that these hyphopodia were aborted.

If the plant accommodates fungal entry by lowering its NT to PACK ratio, especially in its roots, then fungal entry in E151 would have been impeded because that ratio did not decrease enough. At 6 DAI, Jones et al. (2015) noted a significant drop in the NT and PACK levels of E151 plants as a response to the fungal presence; yet this apparently did not lead to fungal entry. We propose that E151 may have overshot the levels optimal for fungal entry and take a longer time than WT to adjust its CK homeostasis. This may reflect an abnormal homeostatic control mechanism (Riefler et al., 2006) during E151 seedling establishment. Later in E151 development, when optimal CK levels are attained, i.e., when an optimal NT to PACK ratio is reached, the fungus would be allowed to enter. This adjustment in CK homeostasis may explain the delay seen in E151.

#### Active CKs Have a Stimulatory Effect on the Intraradicular Growth of the Fungus

Altering CK homeostasis by treating myc^+^ pea plants with synthetic CK status-modifying compounds had a significant impact on the intraradicular growth of the fungus *R. irregularis*. When RB and FB levels increased in the shoots and the NT fraction decreased in the roots and shoots of myc^+^ WT plants treated with INCYDE, the percentages of all fungal structures assessed in the root cortex were significantly higher (Figure 8D). The E151 response to PI-55 in terms of AM colonization confirmed this stimulatory effect. In the myc^+^ mutant, interference with CK perception led to a decrease in all CK fractions, and this translated into a severe reduction of fungal growth within the root cortex (Figure 8C). When E151 myc^+^ plants were treated with a mixture of BAP and PI-55 treatment, intermediate AM colonization was scored, demonstrating that PI-55 and BAP were competing for the CK receptors, and further substantiating the stimulating role of CKs in regulating AM development.

As the levels of PACKs increased (from the levels measured in PI-55-treated mutant, to levels measured in both the non-treated mutant and WT plants, to levels measured in INCYDE-treated WT), there was a simultaneous increase in the percentage of fungal colonization, and all three measured intraradicular fungal structures tended to respond positively to the PACK increase. Based on these data, we propose that active CKs have a stimulatory effect on the intraradicular growth of the fungus, and for higher growth rate of the fungus, the plant requires higher levels of PACKs in its shoots. The plant likely mediates the effects we observed on the intraradicular growth of the fungus because the extraradical fungal growth was not affected in a similar way by the synthetic CK status-modifying compounds. The promoting influence of the shoot CKs on the AM symbiosis that we uncovered fits the model of Cosme et al. (2016) which proposes that shoot CKs have a positive effect on AM colonization. In this model, CKs are thought to modulate carbon supply to the AM fungus through their influence on the plant photosynthetic ability (Cosme et al., 2016).

Our results seem also to agree with those obtained by Schmidt et al. (2017) who performed a pharmacological study of the effect of TDZ, a synthetic CK analog, on AM colonization in the perennial grass *Miscanthus* × *giganteus*. The application of TDZ was found to decrease root colonization by half. This CK analog is thought to be strongly recognized by CK receptors and to prevent CK degradation. TDZ was found to increase the levels of conjugated CKs, but not those of FBs. Upon AM inoculation, the active CK forms were further decreased whereas the conjugated forms were further increased (Schmidt et al., 2017). The low levels of active CKs measured in the *Miscanthus* × *giganteus* grass likely prevented the AM fungi to proliferate intraradically.

#### An Active Role for CKs in the Establishment and Growth of AM Symbiosis Is Confirmed by the Use of the Competitive Inhibitor of CK Action PI-55

There are three known types of plant CK receptors (e.g., Spíchal, 2012; Lomin et al., 2018), represented by CRE1/AHK4, AHK2, and AHK3 in *Arabidopsis*. Each receptor differs in the CK forms it recognizes; thus, CRE1/AHK4 and AHK2 specifically bind FBs and their conjugates, while AHK3 was reported to bind active bases as well as RBs and NTs in a bacterial system (Spíchal et al., 2004). The three receptors likely differ in their functions because their expression is spatially distinct. Whereas CRE1/AHK4 is expressed predominantly in the root, AHK3 is mostly expressed in the shoot, likely sensing the RBs being translocated between roots and shoots (Spíchal, 2012).

In *Arabidopsis*, the synthetic compound PI-55 acts as a competitive inhibitor of CK action, blocking the binding of active CKs to CRE1/AHK4; however, it also interferes with AHK3 (Spíchal et al., 2009). These authors propose that the conformational change of the receptor required for the CK signal transduction pathway to be elicited cannot be triggered when PI-55 binds to it, resulting in a reduced CK status (Spíchal et al., 2009). In pea, PI-55 had been previously used to show CK-antagonizing effect (Long et al., 2012). In the present study, we further showed that PI-55 had a direct effect on CK homeostasis because treating the pea mutant E151 with PI-55 resulted in an increase in NT levels and a decrease in PACK levels, suggesting that the mutant responded to the fully occupied CK receptors by down-regulating its CK biosynthesis, and possibly triggering a feedback loop.

CRE1/AHK4, i.e., the receptor the most sensitive to PI-55 in Arabidopsis, and its orthologs are known to play a major role in controlling nodulation in legumes as was demonstrated in studies performed on the nodulation mutants of *M. truncatula* (*Mtcre1*; Gonzalez-Rizzo et al., 2006; Plet et al., 2011; Ariel et al., 2012), *Lotus japonicus* (*Ljlhk1*; Murray et al., 2007; Held et al., 2014), and *Lupinus albus* (*LaHK1*; Coba de la Peña et al., 2008). However, Laffont et al. (2015), who characterized the response of *Mtcre1* to the AM fungus *Gigaspora margarita*, concluded that CRE1/AHK4 did not play a role in AM colonization. There was no specific mycorrhizal phenotype ascribed to the mutant, and no alterations were observed in the expression of genes related to CK signaling and metabolism in response to the fungus (Laffont et al., 2015). Our results with PI-55 appear to disagree with those of Laffont et al. (2015). This may be related to the different plant and fungal species studied, as well as to the different techniques used to assess AM colonization. Alternatively, it may be because AHK3 is the receptor playing the most important role in AM symbiosis, with CRE1/AHK4 playing a secondary role. AHK3 has a genuine affinity for RBs and for *cis*Z (Romanov et al., 2006). It is likely to sense *cis*ZNT, a CK form significantly reduced in roots of WT and E151 upon AM colonization, because of a better *cis*Z recognition by AHK3 than by AHK4 (Romanov et al., 2006). The AHK3 receptor is also capable of recognizing PI-55 as a weak agonist (Spíchal et al., 2009), and in contrast to CRE1/AHK4, it recognizes free bases with side-chain modifications (Spíchal et al., 2004). Furthermore, AHK3 is expressed moderately in roots and highly in shoots (Higuchi et al., 2004). As we brought forward evidence that shoot CK levels reflect plant mycorrhizal status, the receptors expressed in the shoots could be actively involved in AM regulation.

## Conclusion

The data presented here lead us to propose a stimulatory role for CKs in the development of the pea AM symbiosis and confirm an effect of AM inoculation on plant CK homeostasis. A low NT:PACK ratio was associated with fungal entry and the stimulation of the intraradical development of AM fungi. However, we still face an enigma: i.e., is it the observed decrease in the NT:PACK ratio that allows the extraradical fungal hyphae to enter or is it the intraradical growth of the fungus that is inducing the decrease of the NT:PACK ratio? To answer this question will require more work. Using tissue-specific transcriptome analysis, like Jardinaud et al. (2016) who studied the different roles CKs play in the epidermis and root cortex of nodulated roots, may help solve this conundrum.

## Data Availability

All datasets generated for this study are included in the manuscript and/or the Supplementary Files.

## Author Contributions

FG designed the experiments. DG grew the plants for the cytokinin analysis, performed the developmental study of AM formation, performed all statistical analysis, and made all graphs. SM and ZS performed the pharmacological treatments of the wild type and mutant plants, respectively, and they assessed the fungal colonization of the treated plants. LS provided the synthetic CK status-modifying compounds. AK and RE measured the CK levels and analyzed the CK results. MC and SD performed the pharmacological treatment of the fungus and analyzed the results. MC, DG, and AK participated in many discussions, which led to this manuscript. MC, DG, and FG wrote the manuscript. All authors revised the manuscript.

## Conflict of Interest Statement

The authors declare that the research was conducted in the absence of any commercial or financial relationships that could be construed as a potential conflict of interest.

## Footnotes

1 http://www.mycorrhiza.be/ginco-bel

## Abbreviations

2MeSiP: 2-methylthio-isopentenyladenine
2MeSiPR: 2-methylthio-isopentenyladenosine
2MeSZ: 2-methylthio-zeatin
2MeSZR: 2-methylthio-zeatin riboside
AM: Arbuscular mycorrhiza
ANOVA: one-way analysis of variance
BAP: 6-benzylaminopurine
BAPR: 6-benzylaminopurine riboside
CK: cytokinin
DAI: day after inoculation
DHZ: dihydrozeatin
DHZOG: dihydrozeatin-O-glucoside
DHZR: dihydrozeatin-riboside
DHZRMP: dihydrozeatin riboside-5′-monophosphate
DHZROG: dihydrozeatin riboside-O-glucoside
DMSO: dimethyl sulfoxide
FB: free base
HC: hyphal compartment
HPLC-(+ESI)-MS/MS): high-performance liquid chromatography-positive electrospray ionization tandem mass spectrometry
INCYDE: 2-chloro-6-(3-methoxyphenyl)-aminopurine
iP: isopentenyladenine
iP7G: isopentenyladenine-7-glucoside
iPR: isopentenyladenosine
iPRMP: isopentenyladenosine-5′-monophosphate
MSR: Modified Strullu-Romand
NT: nucleotide
PACK: putatively active cytokinin
PI-55: 6-(2-hydroxy-3-methylbenzyl)-aminopurine
RB: riboside
TDZ: thidiazuron
WT: wild type
Z: zeatin
Z9G: zeatin-9-glucoside
ZOG: zeatin O-glucoside
ZR: zeatin riboside
ZRMP: zeatin riboside-5′-monophosphate
ZROG: zeatin riboside-O-glucoside
