# Supplementary material for: A Stimulatory Role for Cytokinin in the Arbuscular Mycorrhizal Symbiosis of Pea
Source: Front Plant Sci. 2019 Mar 12;10:262. doi: 10.3389/fpls.2019.00262 (PMC6423060; doi:10.3389/fpls.2019.00262)
Supplement: Supplementary file 1 [file Table_1.DOCX]

Table 1. List of the synthetic CK status-modifying compounds used in the pharmacological study whereby endogenous cytokinin (CK) levels were manipulated.

| Compound names | Chemical structure | MW (g) | Function and predicted action | References |
| --- | --- | --- | --- | --- |
| BAP  6-benzylaminopurine | 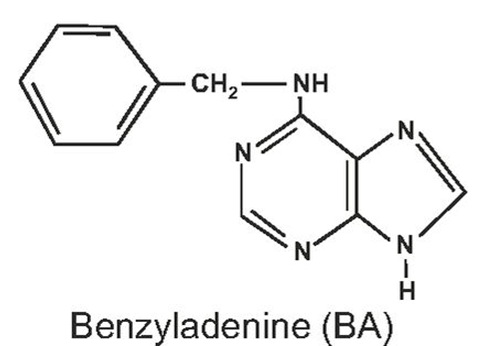 | 255.30 | Synthetic CK –  will increase CK levels | Aremu et al. (2015) |
| INCYDE  2-chloro-6-(3-methoxyphenyl)-aminopurine | 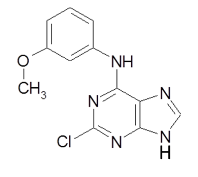 | 275.70 | Inhibitor of CK oxidase/dehydrogenase – will increase endogenous CK levels | Zatloukal et al. (2008) |
| PI-55  (2-hydroxy-3-methylbenzyl)-aminopurine | 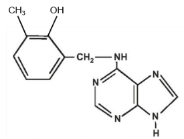 | 255.28 | CK antagonist capable of binding to CK receptors – will decrease CK sensing | Spíchal et al. (2009) |

References

Aremu, A.O., Stirk, W.A., Masondo, N.A., Plačkova, L., Novák, O., Pěnčik, A., Zatloukal, M., Nisler, J., Spíchal, L., Doležal, K., Finnie, J.F., and Van Staden, J. (2015). Dissecting the role of two cytokinin analogues (INCYDE and PI-55) on *in vitro* organogenesis, phytohormone accumulation, phytochemical content and antioxidant activity. Plant Sci. 238, 81-94. doi:10.1016/j.plantsci.2015.05.018

Spíchal, L., Werner, T., Popa, I., Riefler, M., Schmülling, T., and Strnad, M. (2009). The purine derivative PI-55 blocks cytokinin action via receptor inhibition. FEBS J. 276, 244-253. doi:10.1111/j.1742-4658.2008.06777.x

Zatloukal, M., Gemrotová, M., Doležal, K., Havlíček, L., Spíchal, L., and Strnad, M. (2008). Novel potent inhibitors of *A. thaliana* cytokinin oxidase/dehydrogenase. Bioorganic & Medicinal Chemistry 16, 9268-9275. doi:10.1016/j.bmc.2008.09.008

Figure legend for Supplementary figure

Figure 1. (A) Chronology of the experiments performed with plants treated with synthetic CK status-modifying compounds 7, 9, and 11 days after fungal inoculation (DAI). Plants were harvested 13 DAI for CK analysis or 28 (WT) and 35 (E151) DAI for assessing AM colonization. (B) Chronology of the developmental study of AM development in control plants, i.e., not treated with synthetic compounds modifying plant CK status. Plants were harvested at 5 days interval for determining percentages of length colonized by hyphae, arbuscules, and vesicles. Plants used in the developmental study were not assessed for their CK content.
