# Supplementary figures and images for: A Stimulatory Role for Cytokinin in the Arbuscular Mycorrhizal Symbiosis of Pea
Source: Front Plant Sci. 2019 Mar 12;10:262. doi: 10.3389/fpls.2019.00262 (PMC6423060; doi:10.3389/fpls.2019.00262)

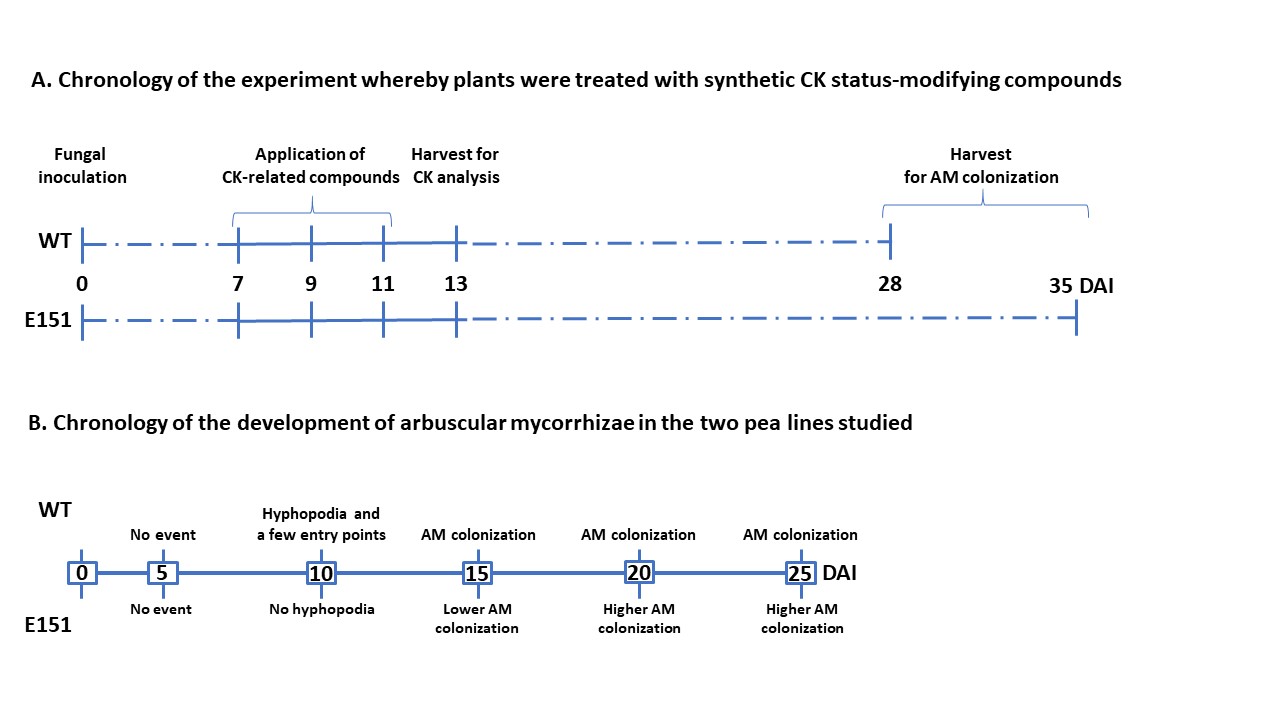

Supplement: Supplementary file 2 [file Image_1.JPEG]
